# Clinical implementation of a bionic hand controlled with kineticomyographic signals

**DOI:** 10.1038/s41598-022-19128-1

**Published:** 2022-08-31

**Authors:** Ali Moradi, Hamed Rafiei, Mahla Daliri, Mohammad-R. Akbarzadeh-T., Alireza Akbarzadeh, Amir-M. Naddaf-Sh., Sadra Naddaf-Sh.

**Affiliations:** 1 Orthopedic Research Center, Ghaem Hospital, Mashhad University of Medical Sciences, Azadi Sq., Mashhad, 91388-13944 Iran; 2 Department of Electrical Engineering, Center of Excellence on Soft Computing and Intelligent Information Processing (SCIIP), Ferdowsi University of Mashhad, Azadi Sq., Mashhad, 9177948974 Iran; 3 Department of Mechanical Engineering, FUM Center of Advanced Rehabilitation and Robotics Research (FUM CARE) and Center of Excllence on Soft Computing and Intelligent Information Processing (SCIIP), Ferdowsi University of Mashhad, Azadi Sq., Mashhad, 9177948974 Iran; 4 Department of Computer Engineering, Center of Excellence on Soft Computing and Intelligent Information Processing (SCIIP), Ferdowsi University of Mashhad, Azadi Sq., Mashhad, 9177948974 Iran

**Keywords:** Biomedical engineering, Orthopaedics

## Abstract

Sensing the proper signal could be a vital piece of the solution to the much evading attributes of prosthetic hands, such as robustness to noise, ease of connectivity, and intuitive movement. Towards this end, magnetics tags have been recently suggested as an alternative sensing mechanism to the more common EMG signals. Such sensing technology, however, is inherently invasive and hence only in simulation stages of magnet localization to date. Here, for the first time, we report on the clinical implementation of implanted magnetic tags for an amputee's prosthetic hand from both the medical and engineering perspectives. Specifically, the proposed approach introduces a flexor–extensor tendon transfer surgical procedure to implant the tags, artificial neural networks to extract human intention directly from the implanted magnet's magnetic fields -in short KineticoMyoGraphy (KMG) signals- rather than localizing them, and a game strategy to examine the proposed algorithms and rehabilitate the patient with his new prosthetic hand. The bionic hand's ability is then tested following the patient's intended gesture type and grade. The statistical results confirm the possible utility of surgically implanted magnetic tags as an accurate sensing interface for recognizing the intended gesture and degree of movement between an amputee and his bionic hand.

## Introduction

Major upper limb loss refers to the limb amputation proximal to the wrist joint, which commonly occurs in healthy young adult males and frequently involves the dominant extremity^1^, particularly at the distal forearm level, where nearly 57% of amputations occur^1,2^. Yet, the current developments in prosthesis design for such cases of amputations come with considerable technological shortcomings. Many upper-limb prosthesis users (35%–45%) continue to object to the technical malfunctioning of their prosthesis systems^3^ in terms of rivaling a human hand and its voluntary and intuitive performance. Clearly, the ideal bionic hand requires significant progress on multiple fronts, including its material, kinematic design, motion generation, energy consumption, sensing technology, and intelligent processing of the sensed information to discover human intention. Such development also requires rigorous clinical testing that investigates the outcomes in the biological human body environment. Moving toward the ideal human-physiologic bionic hand is hence complicated due to the highly interdisciplinary nature of the problem, spanning a wide range of medical and engineering domains. Among them, here we proclaim that a proper sensing technology embedded within the actual biological constraints of an amputee's residual limb and the intelligent processing of the resulting signals are the critical steps in addressing the shortcomings of current bionic hands as a proper alternative to the human hand.

Among various sensing technologies, the electromyogram (EMG)-based approaches with two methods of surface (sEMG) and intramuscular (iEMG) may seem more promising since their signal generation is closer to the source (muscle belly). EMG signals are also more easily attributed to hand motions, at least for cases in which the function of nervous and sensory paths of the amputated limb remains intact^4^. The sEMG approach could control multiple Degrees of Freedom (DoFs) simultaneously through a hybrid regression and pattern recognition algorithm^5^. One successful instance of using sEMG signals uses Self-Organizing Maps (SOM) and Radial Basis Function (RBF) neural networks for classification from surface EMG signals to hand motion, achieving an average accuracy of 96.88 ± 2.73% in recognizing eight hand gesture types^6^. However, sEMG-controlled prostheses are far less competent in determining the 'grade' of motion than a natural hand^7^. The sEMG signals are also an amalgamation of the movement of all the existing muscles (EMG crosstalk) and the noises received from the environment resulting in a non-intuitive and coarse control^8,9^. Furthermore, they depend on ideal electrode contacts, making them susceptible to signal disturbances in practical circumstances^10^. In this regard, iEMG approaches insert the electrodes into the remaining muscles of the amputated area instead of placing them on the patient's skin, resulting in more precision in the performance of the EMG-controlled prosthesis^11,12^. Other less common methods to predict the user intentions make use of electroencephalogram (EEG)^13^, nerve implantation^14^, mechanical vibrations (mechano-myography) while contacting the environment^15,16^, the audio signals created by muscle contraction (phono-myography)^17^, and muscle-generated magnetic fields (magneto-myography)^18,19^.

An ideal sensing technology must be contactless, noise-free, and directly embedded in the relevant actuating muscles. Such signals could be found in magnetic tags implanted in a human hand. In contrast to most previously discussed technologies, in which the sensors are directly in contact with the skin or tissue, magnetic sensors in this technology are simply 'worn' on the skin around the magnetics tags. The movement of magnetic tags along the muscles creates varying magnetic fields that are then used by the sensors to localize the magnets and, consequently, the displacement of muscles. Several studies to date have suggested this alternative sensing technology and simulated the function of magnetic-controlled prostheses^20^. Cipriani and his colleagues performed a series of experiments from 2017 to 2019 on a Myokinetic control interface to study the feasibility of an embedded system^21,22^ using an experimental mockup. They further identified the desired number of magnetic markers by simulating three levels of trans-radial amputation forearms^23,24^. However, these works are tested in electronic hardware only, far from the actual anatomy of an amputee's residual hand. Also, they attempt to control the bionic hand through magnet localization as an intermediate step. Moreover, the relation between magnet location and bionic gestures remains a challenge.

Several studies on this alternative sensing technology detect magnet location^20,25^ through reverse optimization^21–24,26^. Reverse optimization is based on the Dipole magnetic model and does not require regional training; however, neural networks provide more accurate modeling at closer distances to magnets^27^. Magnet tracking over various algorithms, including optimization and recursive ones, is investigated in^28^. Taylor et al. developed an analytical method for multiple magnet tracking that overcomes the optimization algorithms' high latency^29^.

Artificial intelligence tools seem to offer suitable and realistic alternatives to process the data coming from multiple magnetic tags in the complex environment of a human body, environmental noise, disturbances, and motion artifacts. Artificial neural networks (ANN), in particular, are utilized for modeling complex magnetic fields and material behavior^30^ and magnetic field localization^31,32^. ANNs are, in fact, reported to be more accurate in various applications when compared to more conventional algorithms such as non-negative Matrix Factorization (NMF) and Linear Regression (LR), including mapping the hand kinematics from EMG signals^33^. However, they require neural training and are limited to their specific trained regions. Hence, they require proper preprocessing and normalization for better generalization.

In addition to the above challenges, an amputee often misses the relevant muscles. So, a clinical decision must be made to reach a similar degree of motion with alternative muscles. This concern also gives rise to the challenge of intelligent processing by which human intention must be discovered from these alternative muscles' changing magnetic fields. In this new approach, hence, there is a possibility for training the patient to reach higher accuracies, but the resulting muscle-magnet-motion mapping is no longer trivial. In other words, besides the invasive aspect of this technology, it is essential to address the concurrent roles of robotic engineering, artificial intelligence, and surgical orthopedic sciences to reach the desired performance.

Hence, despite its promises, magnet-embedded sensing technology has had no clinical implementation to date. Our suggested approach processes the changing magnetic fields created by the movements of implanted magnets along muscle contractions, termed KineticoMyoGraphy (KMG) signals, for prostheses control. It further offers the first clinical implementation of such magnet technology. It suggests the beginning of a significant modification of prostheses control systems to move bionic hands' function one step closer to its physiological counterpart. The next fundamental issue is the appropriate processing of the detected signal, which is addressed here using a simple neural network with a binary step activation function and a more recent Convolutional Neural Network (CNN). CNNs offer the most significant advantages in reducing the number of parameters with respect to traditional neural networks^34^ by propagating inputs into its hierarchical layers and reaching more abstract features. Moreover, CNN kernels are weight matrices that help reduce noise by simple averaging techniques^35,36^.

From a clinical perspective, this study is also the first to consider the effect of natural forearm biological and physiological conditions (and the adjacent tissues' biomechanics) on the localization performance, because the mockups could never exactly simulate the biomechanical and kinetics of a natural forearm. Besides, prior studies deal with magnet localization, not prosthesis control, as their final goal^22^. Specifically, we report on the design and development of a KMG-controlled bionic hand and its clinical implementation on a volunteer patient as an unprecedented step in advancing the field of magnetic-based prostheses (please see Supplementary Movie S4). More specifically, the first clinical trial of implanting magnetic tags into the forearm muscles of a distal forearm level amputated patient using the tendon transfer operational technique is reported here. The magnetic signals from the implanted tags form the KMG signal that drives the prosthesis hand by detecting gesture types and grades of motion using artificial neural networks. The procedure maps the KMG signal extracted from moving magnets directly to the bionic hand gestures, skipping the magnet localization step. In other words, our strategies are an alternative approach that combines two steps of magnet localization and intention detection into one. Analyzing the relation between the contraction/expansion of the muscle with the up/down trend of the KMG signal reveals an intuition similar to the physiological hand.

**Figure 1:**
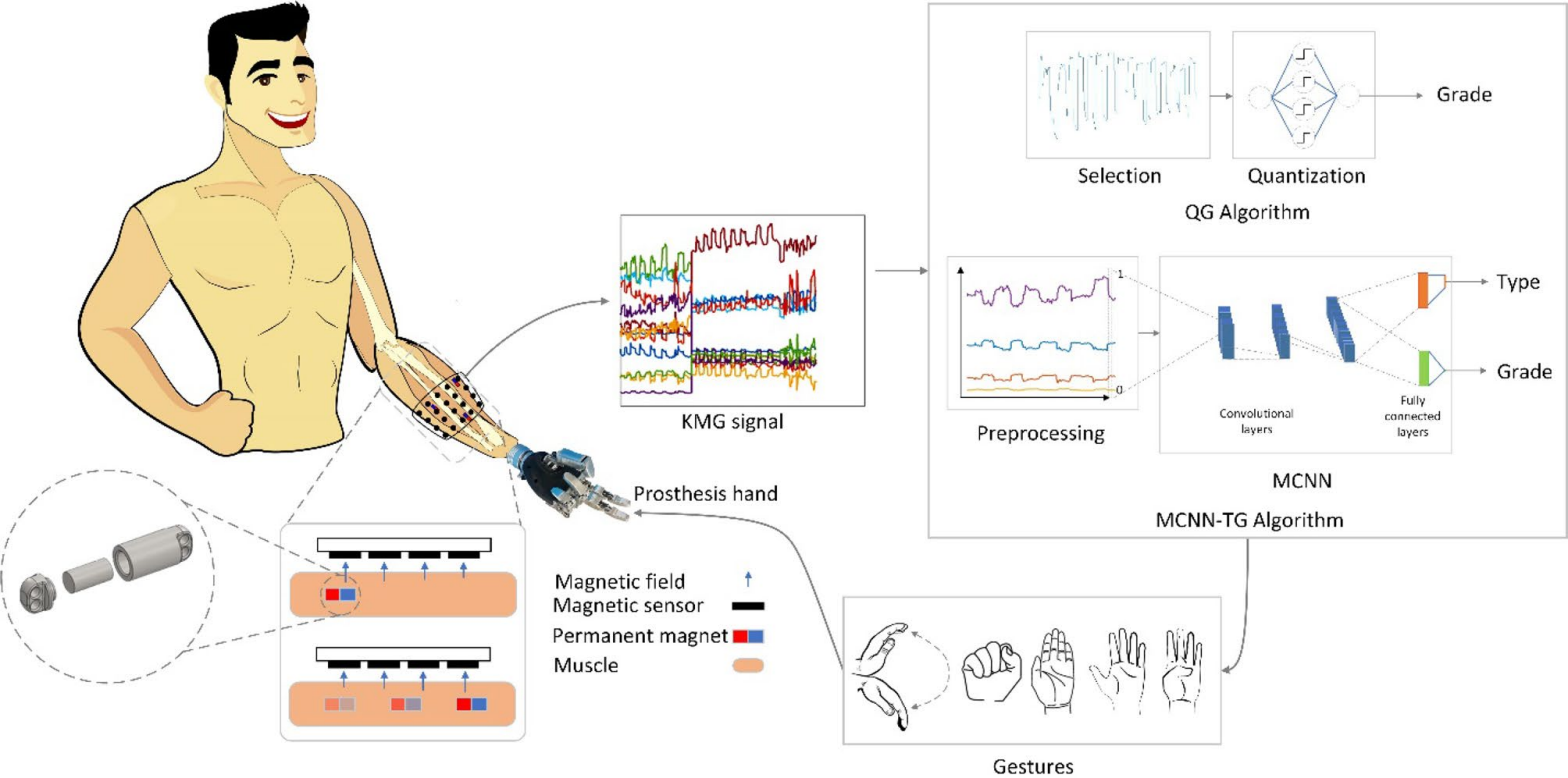
The general structure of the prosthesis hand controlled by the KMG signals. The number of KMG signal channels generated from the implanted magnets is proportional (one channel for each Cartesian coordinate) to the number of sensors. This signal is used to directly drive the prosthesis.

**Figure 2:**
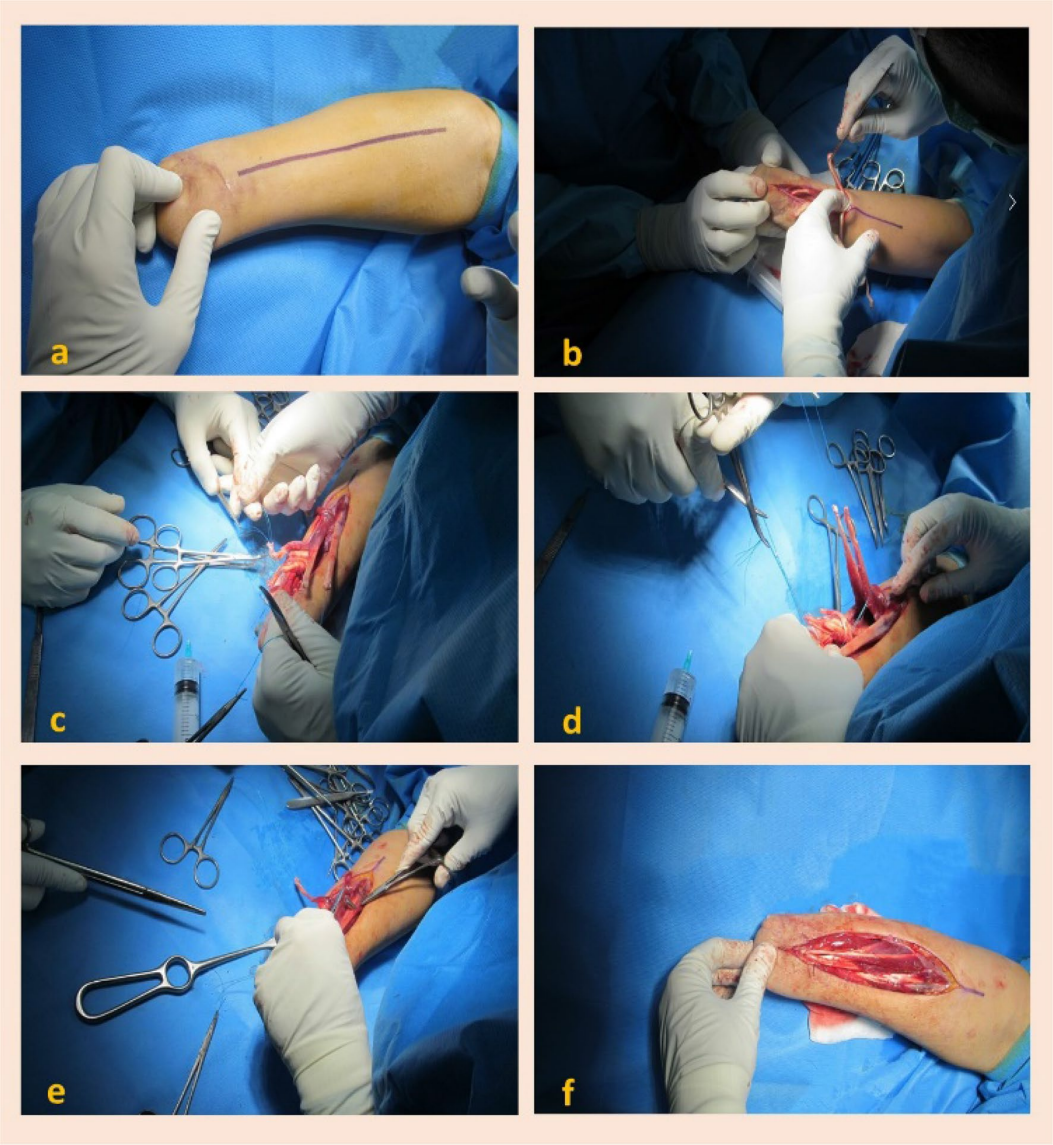
Tendon transfer operation technique. The skin and subcutaneous tissue are exposed (**a**). The viability and function of each pair of muscles are assessed with the cautery electric stimulation (**b**). Corresponding to each target muscle in the volar, the dorsal muscles are selected (**c**). Then, along with the distal third of the forearm, volar and dorsal aspects are exposed to reach the interosseous membrane, after which the flexor tendons are transferred to the corresponding extensor tendons under moderate tension (**d**). Magnetic tags are placed in the most superficial flexor level inside the musculotendinous junction (**e**). Skin and subcutaneous tissue repair (**f**).

Figure 1 shows the overall structure of our proposed bionic hand. The first step of our research includes inserting magnetic tags in pairs of synergic forearm muscles responsible for cardinal hand movements using the tendon transfer technique (Fig. 2). Following the voluntary movement of the residual muscles, the implanted magnets also move. These magnets create KMG magnetic signals that are measured by an array of magnetic sensors. In order to convert the KMG signal to the desired hand movement, we have designed two neural algorithms. The first Quantized Grade (QG) algorithm uses only one magnet and, therefore, only one gesture. If chosen, the bionic hand should be manually switched to different gesture types in this algorithm (please see Supplementary Movie S1). The QG algorithm was validated using a rehabilitative Fist and Ball game that was developed in three levels: simple, moderate, and advanced. During the game, the patient tries to grab the bouncing ball. The second Multi-target Convolutional Neural Network-Type and Grade (MCNN-TG) algorithm identifies both the type and grade of gestures based on the value of the multiple magnets' movement. Min–max normalization is applied to all KMG signal channels at each time step to improve the MCNN-TG learning and prepare the same scale features (KMG signal channels). Therefore, the maximum magnetic field reported by each sensor in each sample is limited to one. Finally, the prosthesis hand performs the desired movement as determined by the neural architectures.

## Results

### Operation and the post-operation fluoroscopy

Three pairs of muscles with higher tendon excursion (meaning the distance a tendon travels upon muscle contraction) are transferred using the tendon transfer technique (Fig. 2), and one magnetic tag is implanted in the flexor component of each transferred pair under fluoroscopic guide (Table 1). Fingers, wrist, and thumb flexion/extension correspond to tags one, two, and three, respectively. These fluoroscopic records are then archived for further follow-up comparison. At the end of Weeks 3 and 12 postoperatively, the patient's forearm is imaged under fluoroscopy while he is requested to perform three different hand and wrist virtual movements (fingers, thumb, and wrist flexion and extension), considering three transferred muscles' functions. The maximum movement is then observed in tags one and two (5.7 and 4.7 mm after 12 weeks, respectively), while the third one does not have a significant independent movement (Table 1). The fluoroscopic patterns of tag displacements for each patient's hand movements are different, thus assisting the classification of each movement signal.

**Table 1:** Transferred muscles data and tag displacement outcomes.

	Tag one	Tag two	Tag three
Flexor muscle	Flexor digitorum profundus	Flexor carpi radialis	Flexor digitorum superficialis
Extensor muscle	Extensor digitorum communis	Extensor carpi radialis brevis	Extensor pollicis longus
Rehabilitation	Grasp and release	Wrist flexion and extension	Thumb extension and fingers flexion
Independent movement	Yes	Yes	No

Type of hand movement	Tag displacement (mm) (week 3/week 12)		
Finger flexion and extension	4.46/5.7	1.2/1.7	0/2.4
Wrist flexion and extension	2.8/3.3	3.1/4.7	2.1/2.5
Thumb flexion and extension	1.5/0.7	2/1.8	3.2/3.2

### Control of prosthesis hand by QG gesture detector algorithm

**Figure 3:**
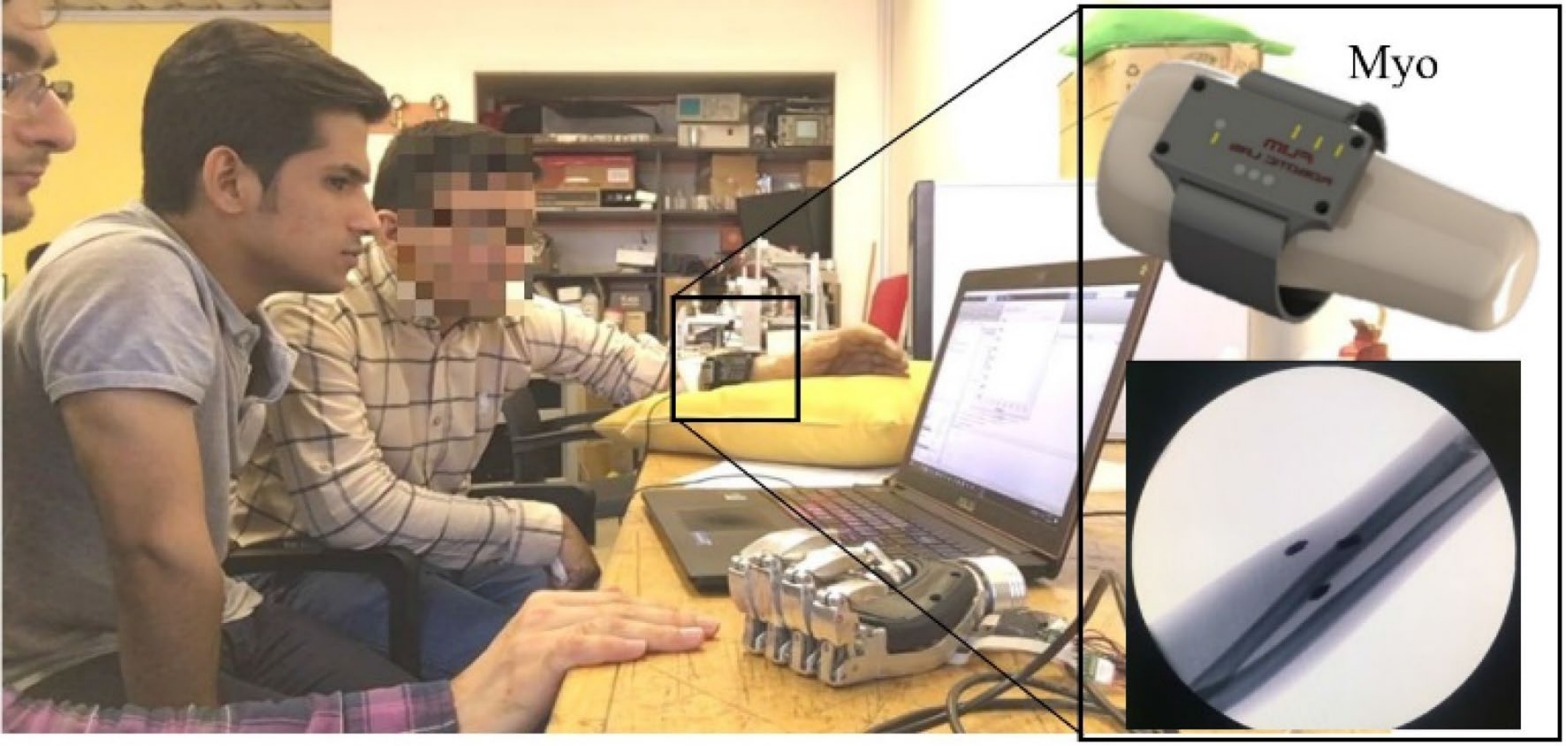
Connection of the prosthesis to the Myo along with an x-ray that shows the placement of magnets inside the patient's amputated arm. The measured magnetic fields by the Myo sensors are immediately transferred to a computer. Processing the measured signals is carried out by MATLAB software to map the magnetic field to the bionic hand movement. These movements are then fed to the present bionic hand motors for the appropriate gesture. In this picture, the patient has voluntarily closed his prosthesis hand. The participants gave written consent to include their images in this article.

After ten weeks, mounting a prosthesis on the patient's amputated limb is examined to show the QG algorithm's feasibility. In this first algorithm, the KMG signal ranges are partitioned into nine quantized grades (Fig. 3). The associated results are presented in the supplementary video file (Movie S1) in which we have shown the patient to fully and partially flex and extend fingers and hold the partially flexed position. Also, the game rehabilitation provides further results of this algorithm.

### Intention detection by MCNN-TG gesture detector algorithm

The second designed algorithm includes identifying both the type and grade of movements. Overall, 7000 samples are collected for each gesture simultaneously with the prosthesis mounting. As mentioned in the QG algorithm experiment, the data samples consist of 18 dimensions (16 dimensions for input related to the 16 sensors amplitudes, one output for the gesture type, and one output for the motion grade). The first 80% and the remaining 20% of the dataset (KMG signals) are used for training and testing, respectively. It should be noted that QG's algorithm is tested in a real-time setting, while MCNN-TG's training and testing at this point are both on the recorded dataset due to the algorithm's computational cost, the realistic limitations of processors on a bionic hand, and the resulting delay. The MCNN-TG network consists of three packs of two layers, including one convolutional and one batch normalization, following two fully connected layer heads: gesture type and gesture grade. The network is trained using ADAM optimizer over 500 epochs with a learning rate, gradient decay, and squared gradient decay of 0.001, 0.9, and 0.999, respectively. Table 2 shows the proposed algorithm's accuracy and error for estimating the gesture type and grade.

**Table 2:** Accuracy of estimated gesture type and grade error in different motions (wrist extension/flexion (I), clenching (II), and thumb extension/flexion (III)).

Gestures	Gesture type	Gesture grade^a^			
	Accuracy (%)	RMSE (°)	EMR (%)	MAE (°)	MRE (%)
Motions I and II	100	5.8	$\approx$ 8	11.27	3.1413
Motions I and III	100	4.06	$\approx$ 5.7	6.24	1.3236
Motions II and III	100	6.9	$\approx$ 8.6	10.34	1.3544
Motions I, II, and III	100	7.26	$\approx$ 10.3	15.3	4.1626

RMSE, root mean square error; EMR, error in movement range; MAE, maximum absolute error; MRE, mean relative error.

^a^Gesture grade is the stage of the motion completeness in a range between 90 to 180 degrees.

### Rehabilitation game exercise by QG gesture detector algorithm

The patient performed a designed rehabilitation exercise, "Fist and Ball game", using the same prosthetic hand controlling QG algorithm in three easy, intermediate, and advanced levels. We ran the game two times in the laboratory, separated by one week. During this one-week interval, between the two tests, the patient repeatedly performed several tests himself to rehabilitate. The repeated test results showed relative improvement in performance. The difficulty level (mode), ball speed, number of up and down movements during total running time, duration of time the patient held the ball, and the final score are presented in Table 3.

**Table 3:** Rehabilitation game exercise results.

Mode	Experiment parameters		Performance metrics (first test result-second test result)		
	Speed (m/s)	Up and down movements (num)	Total time (s)	Holding time (s)	Score^a^
Simple	5	2	84–44	26–39	31–88
Intermediate	8	17	67–58	23–49	35–84
Advanced	20	16	91–41	48–27	52–65

^a^Score is calculated with the following formula:$(\mathrm{holding time}/\mathrm{ total time})\times 100$

## Discussion

Here we consider the KMG signal and its use as part of a hand prosthesis from the aspects of the physiological parameters, operational methods, signal representation, mobility range, noise sensitivity, and intuitiveness. We also discuss the gesture detection shortcut, classification accuracy, and control delay. Future aspects of this technology are then addressed, such as its noise and disturbance rejection, the feasibility of multi-gesture control, and optimization-based methods.

The clinical implementation in this study indicates that the inserted tag in the first pair of muscles, where the fingers flexor and extensor are transferred, displays the greatest range of motion, compared to the two other pairs of muscles. This finding, while preliminary, suggests that applying finger flexor and extensor tendons, which have the highest excursion (meaning the distance a tendon travels upon muscle contraction) among three pairs of transferred tendons, are the proper sites for tags to be inserted. This is because muscles with a higher excursion give us the chance to increase tag movement. Therefore, if we had transferred all flexor digitorum profundus tendons to the corresponding extensor tendons as a pair, more forceful contractions would be available through the insertion of a single tag into the several synergic muscles transferred together. Unfortunately, though, the resultant movements of tags are actually less than expected. These under-expected results are likely to be related to the tendon transfer method used and the secondary tissue adhesions, which affect both dependent and independent tag movements. The results could be improved by: (1) administering an anti-adhesion agent, (2) applying a non-adhesive artificial furculum instead of the interosseous membrane, and (3) application of hunter rods to create a tunnel for smooth tendon gliding. It is possible to distinguish three independent patterns of tag movements using rehabilitation exercises; however, in our study and after 12 weeks of rehabilitation, tags one and two movements became independent while tag three did not move independently. That is because the movement pattern of its corresponding flexor digitorum superficialis and extensor pollicis longus muscles contract to some extent following the contraction of the other two transferred pairs of muscles. It also seems possible that if we simply suture the distal end of the tendon to the subcutaneous skin under appropriate tension, we would have similar results with less aggressive procedures. We did not measure the displacement of the magnets in relation to the suture positions, precisely. However, in our serial follow-up radiographies with the forearm in relaxing posture, the tags were at the same relative position, when comparing the serial images. Besides, the movement of the tags was not disrupted for one year. As such, we can say that no tendon, muscle, or suture rupture was observed.

The proposed KMG representation differs from the conventional dipole optimization and neural network-based approaches that use the magnetic field components in the three-axis. Our proposed approach uses the magnetic field's magnitude, leading to simpler network architectures and more generalization. Specifically, we tune the QG algorithm manually once and examine it by running three experimental modes twice, with a minimum of one-week interval. During these three modes, forearm muscles do not move similarly, and Myo does not stay in the same position. We observe an improvement in the second rehabilitative game examination without any performance drops, indicating that the algorithm is robust to Myo displacement.

Nonetheless, the range of the implanted magnet (in the muscles) changes over time. Due to surgery complications, the inserted magnets initially have low mobility; however, as time passes and more exercises are performed, the magnets' range of mobility improves, contributing in two ways. High mobility range enables us to use sensors with lower sensitivity and achieve a higher signal-to-noise ratio. The increased mobility of the magnets also facilitates the identification of gesture type and grade. Based on the mentioned surgical results, we concluded that the KMG-controlled system could derive the intentional muscular movements since different tag movements result in different patterns to be identified and processed in the bionic hand-control systems.

The EMG control approaches are also highly challenged in terms of their comfort and simplicity of usage for long durations, intuitive performance among amputee patients, and a limited number of electrodes^7^. Anatomical factors, biomechanical factors, electrode structure and placement, and signal noise configure the EMG signal. The EMG signal is affected by various noise sources, including the inherent electrical noise, ambient noise, motion artifacts, and signal instability. For robust gesture recognition, EMG signals should reach the highest informational signal-to-noise ratio and minimum distortion^37^. Accordingly, for robustness analysis, we calculate the SNR for KMG signal and EMG as the most commercially used signals for bionic hands. The EMG dataset is obtained from^38^, and we calculate the SNR of the two signals using the built-in *snr* function of MATLAB software based on sampling frequency (3000 Hz for EMG and 42.7 Hz for KMG) and periodogram power density estimate. The computation of noise here excludes the power of the first six harmonics, including the fundamental. Results indicate that the SNR for EMG could be as low as -19.5 dB, while it is 4.17 dB for the KMG. Therefore, the KMG signal offers more robustness in gesture recognition. Notably, these results here come from the direct application of KMG signal without denoising or preprocessing filters.

**Figure 4:**
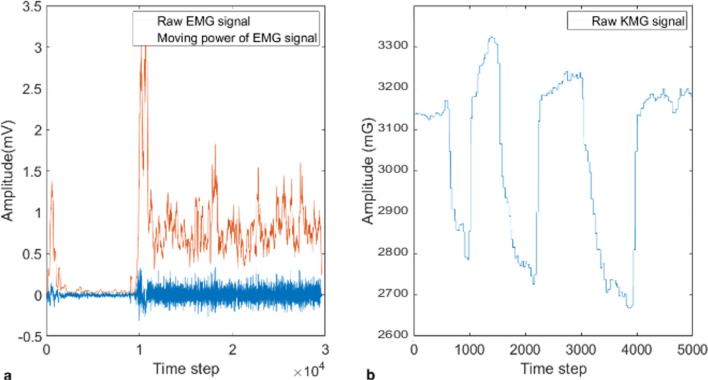
Both data sets are related to the wrist flexion and extension of corresponding muscles. The EMG signal is adopted from ^39^ related to sensor six adjacent to the extensor Carpi Radialis muscle, subject 4, and fourth test for 10 s at a frequency of 3000 Hz (**a**). The KMG signal is recorded from a sensor close to the wrist magnet implanted in transferred tendons of flexor Carpi Radialis and extensor Carpi Radialis Brevis muscles for 117 s at a frequency of 42.7 Hz (**b**).

This designed bionic hand is unprecedented in its control method, which uses magnetic tags in the muscles. As illustrated, the structure of the KMG-controlled prosthesis is both undemanding and economically more efficient than other existing methods since it needs several magnets and magnetic sensors at a lower cost than other methods' electrodes. Furthermore, the analysis of the KMG signal shows a consistent behavior (peak and bottom) with the contraction/extraction of the muscles and, subsequently, with the intuition of the amputee (Fig. 4). For example, when the amputee is clenching, the amplitude of a magnetic field raw signal reported by a sensor visibly increases, but such perception is not realized from EMG raw signals. Also, on–off control methods are based on a threshold to activate the bionic hand and are not completely compatible with the morphological feedback sensed from residual muscles. In our proposed position control, the bionic hand movement is coordinated with the muscle contractions/extensions and thus is more intuitive.

Here, we derive the control signal without the magnet localization as an intermediate step to gesture detection. Magnet localization provides helpful information about muscle and magnet movements during various gestures. However, the ultimate goal of muscle movement detection is the bionic hand's proper control. Therefore, this research focuses on end-to-end control of the bionic hand using raw signals received directly from sensors. Specifically, the QG algorithm uses the tags’ magnetic fields to determine the movement and position of the bionic hand, bypassing the magnet localization step. Also, in the MCNN-TG algorithm, thanks to the progressive feature extraction abilities of the deep learning approaches, all the needed features (maybe a feature about magnet locations) to determine the intended gestures and their degrees are automatically found by the network itself.

Recent studies have noted the accuracy of gesture classification using EMG signals. One study shows 72.19% accuracy based on the formulation of Few Shot Learning^39^. Another paper led to 96.875% accuracy in the recognition of eight gestures based on self-organizing maps and radial basis neural networks^6^. For EEG signals, the highest reported accuracy is 80% for vertical upper limb movement, using a deep learning algorithm trained with window sizes of 600 ms^40^. These approaches are evaluated under various amputee hand situations and testing conditions. The suitability of the bionic hand control mechanism, in our opinion, is case-specific and should be determined on an individual basis. As a result, rather than relying solely on accuracy, a fair comparison requires numerous criteria. During testing the dataset, however, we achieved 100% accuracy in three gesture classifications utilizing the KMG signal (Table 2).

The optimal delay in EMG-based prosthesis systems is between 100 to 125 ms^41^. Surface High-Density ElectroMyoGraphy (HD-EMG) and a CNN trial led to a latency of 116 ms in posture prediction in an embedded coding^42^. In SOM and RBF networks, the response time is reported as 430 ms^6^. In our study, the implanted magnetic tags move along with the muscle contraction; thus, the resultant KMG signal represents the muscle movements. The QG Gesture Detector algorithm has a simple architecture and can be executed with a small delay. We run the QG algorithm on a PC (Core i7 4720 HQ 2.6 GHz, Ram 16 GB, Nvidia 960 m—4 GB) to transfer 7000 samples, where the average transfer delay from sensors to the Blender software execution was 41.6 ms per sample. The MCNN-TG gesture detector algorithm may need more processing time to determine the output and is not measured in this work. For the QG algorithm, we measure the total time from sensing the raw signals to bionic hand gesture detection, while recent studies report only the localization of 5 magnets in about 4 ms via optimization approaches. The above localization and gesture detection algorithms are executed on different hardware and serve different purposes. Hence rather than being competitive, their hybrid may lead to a faster and more efficient algorithm for gesture detection.

Furthermore, to highlight the high KMG signal quality, the proposed intelligent approaches in this work are examined without the usual preprocessing steps such as signal cleaning, outlier detection, and noise cancellation. The disturbance effects are suppressed using thresholds and convolutional layers. Therefore, the geomagnetic field as a permanent source of contamination (geomagnetic field changes are sufficiently smaller than KMG signal changes) could be compensated; however, the effects of varying environmental magnetic fields on the performance of prosthesis hand control should be investigated using adaptive filters and noise cancellation methods. Also, further analysis should be performed to examine the robustness of the proposed algorithms to the environmental noises and disturbances, magnetic effects of the prosthesis hand, and magnets' rotational movements.

In this study, the control algorithms determine movements for a single gesture at each time step. The proposed methods could be extended to simultaneous combined gesture detection for prosthesis hands. The QG algorithm achieves this goal by having multiple parallelized QG algorithms for multiple gestures using multiple sensors, provided that there is an independent movement of magnets for each gesture. In contrast, the MCNN-TG algorithm needs a structural modification for concurrent detection of gestures.

As mentioned earlier, optimization-based magnet localization needs no training and can be transferred across patients and setups. But similar to the proposed approaches in this research, these techniques also require training to determine hand gestures, considering that magnets' location and movements differ from one patient to the other. Here we address the end-to-end control of the prosthesis hand; hence, our algorithms need to be adjusted for new patients. Further study should be performed to compare the control strategy based on the two-step process of optimization methods and the proposed end-to-end neural networks for prosthesis hands control systems.

In the future, modification of surgical procedures regarding tendon selection for transferring and tissue adhesion is recommended to achieve more robust tag movements and, therefore, more powerful signals. We also hope to investigate further the bionic hand's control system based on the separate movements of each finger and detecting multiple simultaneous finger movements. To do so, we would need to determine concurrent finger activation instead of gesture types. Because the regression performance cannot be measured in real-time due to lagged appearance of supremum, a challenge may arise in adaptive structures that we hope to address in future works. Furthermore, the positive results of this paper guide us in studying the KMG signal for the bionic hand's dynamic speed control. We plan to investigate muscle changes in both thickness and length. When the patient freely moves his phantom limb, muscle length is mostly affected while muscle thickness changes in power motions^43^. Furthermore, we want to develop embedded processing hardware to address the extra computational cost of MCNN-TG and the resulting delay in its real-time implementation. Finally, we also hope to investigate the utility of this method for lower limb amputees.

## Materials and methods

This case study was conducted at Mashhad University of Medical Sciences (MUMS) in collaboration with the Center of Advanced Rehabilitation and Robotic Research of Ferdowsi University of Mashhad (FUM)^32^. More specifically, the design and manufacturing of tags and the prosthesis as well as the development of the algorithms and game exercises were carried out in the FUM Robotic Center, while the surgery and the patient's follow-up visits were performed in Imam-Reza hospital, Mashhad, Iran. This study was approved by the Research Ethics Committee of Mashhad University of Medical Sciences (approval number: IR.MUMS.MEDICAL.REC.1398.099) and is conducted in full compliance with the codes of ethical conduct from the 1964 Declaration of Helsinki. Regarding Fig. 3, we confirm that informed consent was obtained to publish the images in an online open-access publication.

This section is divided into two parts of (a) medical procedure (surgery), and (b) engineering. More specifically, we continue by explaining surgical procedures of magnet implantation, the proposed QG and MCNN-TG neural networks, Myo and Socket construction, and finally, the structure of the game therapy.

### Medical procedure

Before initiating the surgery, the engineering team designs, manufactures, and sterilizes the magnets. The neodymium magnet could not be implanted in the patient's body without being covered because it is easily eroded and cause after-effects. Therefore, these magnets are covered with biocompatible materials. Medical steel 316, which also does not cause any distraction in the magnetic field, is used as a bio-compatible material for covering the magnets.

The standard range for magnetic fields inside the body (medical implant) is 0.5 mT^44^. One well-demonstrated biological impact of magnets on the body is increased blood circulation in tissues. Although, at very high frequencies, it can cause thermal damage^45^. This study's calculation is based on magnetic flux, capsule thickness, and magnet size, which comply with the standard range. The resulting tags are cylindrical with 13.3 mm in length and 5 mm in diameter, weighing 1.1 g. The magnets are first placed into a sterilized capsule to be welded by laser, after which the capsule is electro-polished and is finally sterilized with plasma rays.

To find the patient who fulfills our inclusion criteria, we reviewed the medical information of patients suffering from forearm or wrist amputation, which had occurred between 3 to 12 months prior to this step, across three trauma centers. Criteria for selecting the patients are the following: between 18 and 60 years of age, psychologically stable, a full range of motion in shoulder and elbow, unilateral upper limb amputation between the distal and middle one-third of the forearm, and finally, high motivation for rehabilitation and using artificial hand. Patients with immune deficiency, diabetes, kidney disorders, extensive skin scar, and those whose amputations are secondary to a tumor are excluded. Initially, patients were contacted and informed about the study, and among the eleven candidates, four volunteers accepted to participate in a discussion session with our senior hand surgeon.

Accordingly, a 28-year-old man with a history of radiocarpal joint level amputation from four months earlier agreed to participate in the study and subsequently signed the consent form (Attachment 1), once the hand surgeon had explained the details of the surgery to him. Here, the process of this surgery is explained in three steps: pre-operation, during operation, and post-operation.

#### Pre-operation

Once the patient is enrolled, a para-clinic study is carried out using bilateral forearm MRI and EMG-NCV to identify the fatty change of forearm muscles and neuromuscular conditions, respectively. To rule out the possibility of a foreign body's existence and evaluate the bony structure, the patient's forearm undergoes X-ray studies. In the case of this patient, we did not detect non-functional muscles, severely atrophic muscles, or any foreign objects.

#### Operation

This surgery aims to implant at least three magnetic tags in three pairs of synergic flexor–extensor forearm muscles, which are responsible for cardinal hand movements. For this purpose, the tendon transfer method was chosen as the surgical technique. Three pairs of muscles are considered to be transferred (Table 1). The procedure is performed with the patient in the supine position following induction of general anesthesia without using anticholinergic drugs. The arm was then prepped and draped. Using two separate longitudinal dorsal and volar incisions (Fig. 2a), the forearm's remaining flexor and extensor muscles are exposed and released. Each muscle in the volar and its corresponding dorsal muscle was isolated and stimulated with electric stimulation to assess the viability and function (Fig. 2b). Then, along with the distal third of the forearm, volar and dorsal aspects were exposed to reach the interosseous membrane. Distal five centimeters of the interosseous membrane were released from its bony attachments and rolled up proximally to function as a fulcrum for each pair of muscles. Using the Pulvrtoft suture technique, the flexor tendons were transferred to the corresponding extensor tendons under moderate tension (Fig. 2d). Moreover, we try to place the suture lines far from the fulcrum. Then, magnetic tags are placed in the most superficial flexor level inside the musculotendinous junction (Fig. 2e), and are fixed using four separate 4–0 nylon sutures (Fig. 2f). Movements of the transferred pairs of muscles are checked using electrical stimulation. Before closing the cutaneous and subcutaneous layers, re-imaging is performed to ensure the correct position of the tags by fluoroscopy. Fluoroscopic films are recorded and saved for future follow-up comparison. Finally, compressive dressings were applied. Figure 2 represents the steps of the operation.

#### Post-operation

After the surgery, the patient was administered antibiotics (Cefazolin) for 48 h, and then discharged. At the end of weeks one and two postoperatively, follow-up visits were done to evaluate the probable early surgical complications or wound problems. During every visit, a control X-ray radiography was performed to assess the tag positions. After two weeks, the post-operative sutures are removed, and the patient began rehabilitation physiotherapy sessions while still on a compressive bandage to prevent edema. Three weeks after the operation, during another fluoroscopy, the patient was asked to move the muscles actively under radiation shield protection, and we recorded the amount of displacement of the tag movements. (Table 1 and Supplementary Movie S2).

By week three, the FUM robotic team successfully developed an electronic Myo with nine LEDs determined in three different color bars, each of which includes three LEDs that could detect the tag movements. The Myo is designed to (1) increase the range of motion of the tags, (2) increase control over the performance of the movements, (3) find out a specific pattern for every single hand movement, and (4) rehabilitate the atrophic muscles by giving the patient visual feedback leading to more motivation. The patient was then asked to frequently perform certain exercises in both hands at home during the next six weeks to rehabilitate his muscles (real movements with the normal hand and imaginary ones with the amputated hand) (Table 1). At the end of week 12 postoperatively, the patient's hand was imaged by fluoroscopy for the second time in two directions, which were perpendicular relative to each other (Table 1 and Movie S3). In order to measure the tag displacements, the fluoroscopic films are used to determine the moments when the tags had maximum displacement, which is then compared with the neutral position.

### Engineering design

Our engineering efforts are divided into four main steps: data preparation, intelligent processing, implementations by designing appropriate Myo and Socket apparatus, and patient training in using the hand by designing a game. It begins by describing how data is prepared for classifying hand gestures and grades. It will then continue with intelligent processing algorithms, including one shallow and one deep artificial neural network. The shallow network is a one-hidden layered feed-forward neural network that is intuitively tuned for performing as a quantizer (the act of transforming the continuous quantity of magnetic field to certain discrete grades). This network provides the basic desire for manipulating the prosthesis by gradually opening or closing it. Furthermore, for obtaining complex tasks like gesture recognition, we developed a Multi-target CNN with several convolutional layers followed by fully connected layers trained to simultaneously determine the intended gesture types and grades.

Subsequently, we design a Myo and Socket consisting of three and sixteen magnetic sensors, respectively, to implement our data collection method. Finally, we develop a therapeutical rehabilitation computer game for enhancing and restoring control skills over the prosthesis hand.

The MATLAB software performs all the procedures of signal acquisition, monitoring, signal processing, designing the algorithms, training the networks, and the control signal generation. The Blender software and Python programming language are used for simulation of the hand and the designed rehabilitative game.

#### Preparing the data

In this section, we provide the procedure of data preparation for both algorithms.

*(a) QG algorithm*

Having inserted the magnets in the patient's forearm, the KMG-based approach can then be studied. Following the displacement of magnets, the resultant magnetic field is measured by the sensors. First, the sensor with the most value variation is selected, and its value is quantized to several grades. Next, the full range of prosthesis hand gestures (180°) is divided into an equal number of the first step grades. Therefore, we have a certain position of the prosthesis hand for each magnetic field interval.

*(b) MCNN-TG algorithm*

We asked the amputee to perform the specific motions orally without pause, including finger flexion and extension, thumb flexion and extension, and wrist flexion and extension. The amputee performs each of the three movements at least ten times at a constant rate. Then, the implanted magnet's magnetic field components are recorded using magnetic sensors with a sampling frequency of 42.7 Hz. The amplitude of magnetic fields is calculated for further processing as follows:

$${\varvec{B}}=\sqrt{{B}_{x}^{2}+{B}_{y}^{2}+{B}_{z}^{2}},$$

where ${\varvec{B}}$ is the amplitude and ${B}_{x},{B}_{y},{B}_{z}$ are the components of the magnetic field. Therefore, the number of KMG channels equals the number of apparatus sensors. Then, a label is assigned to every movement for further classification purposes. Also, among the data received by the sensors, those data with the smoothest pattern and most variation are selected for each movement. For each gesture, sensors with the greatest peak-to-peak amplitude are selected to account for full extension and flexion of the hand. After that, we visually select the sensor with the smoothest pattern. Then, according to the maximum and minimum amount of signals, a certain sinus shape pattern of motion range (180°) is ascribed to every movement using a smooth function for regression purposes. In this fitting procedure, every two consequent extremums of the KMG signal are connected by a smooth curve. This procedure is applied to training and testing data. The performance measure in testing for this algorithm is calculated after passing each extremum. The obtained dataset is directly fed to the following networks without feature extraction. The networks determine gesture type and grade based on the moment data recording, without the inclusion of window data. In short, the MCNN-TG algorithm learns gesture types and related degrees based on the magnetic field of moving magnets.

#### Quantized grade (QG) gesture detector

To quantize each hand movement, we defined gesture grades. According to this definition, each grade represents a unique position during the movement. Following the voluntary displacement of the magnets, the resultant norm of the magnetic field is mapped to the grade of the bionic hand movement for a specific gesture. The mentioned procedure is expressed in a simple neural network with a binary step activation function in a hidden layer and weights to determine quantization grade. The hidden weights are used to scale the input and integer output weights are specified grades. The network weights are completely determined by trial and error, and there is no need for any training procedure for this algorithm.

#### Multi-target convolutional neural network—type and grade (MCNN-TG) gesture detector

In this study, we use a multi-target convolutional neural network for two different purposes: gesture type classification and motion grade determination. The KMG signal enters the convolutional layers and then passes through two fully connected parallel layers for classification and regression targets. Therefore, the bionic hand movement grade could be estimated in addition to identifying the gesture type.

#### Designing and constructing the Myo for the QG algorithm

Once the magnetic tags move inside the body, the magnetic fields measured by the designed multi-purpose Myo sensors (LIS3MDL) from STMicroelectronics Inc. transmit this data to the computer (Fig. 3). In this gadget, magnetic fields are measured using an array of three magnetic sensors connected to an Arm Cortex-M3 processor by I2C connection. The information received is then sent to the computer by the ESP8266 module to be processed in MATLAB software, thus determining the specific hand movement corresponding to the patterns of the magnetic field. Since the QG algorithm needs one single magnetic sensor, we only use data from the array's middle sensor. The intended movements are then transmitted to the artificial hand motor, and the artificial hand performs the movement.

#### Rehabilitation game exercise

**Figure 5:**
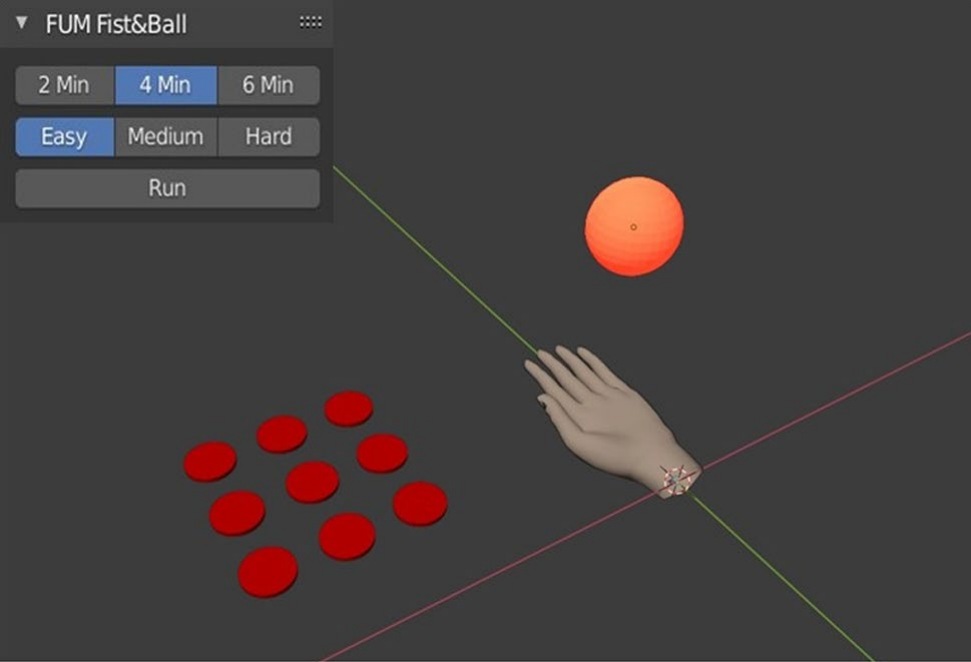
Simulation environment of Fist and Ball. It consists of nine red plates as LEDs of the electronic Myo, a 3D model as a hand, and the orange plate as a ball. The left upper panel shows the control panel of the Fist and Ball environment with three exercise durations and difficulty levels.

The primary goal is to provide a user-friendly rehabilitation environment for the patient that can be run on his own PC. In this way, the muscles are strengthened and the internal stickiness between tissues is reduced, because the tags' free and independent mobility remarkably affects the control performance. The secondary goal is to provide an illustration environment for evaluating the performance of this study's various gesture estimation algorithms. In order to assess the patient's performance, we implement a rehabilitation game exercise 12 and 13 weeks' post-op whose objective for the patient is to catch and keep the ball which is moving up and down with the 3D model of a hand in a virtual environment through movements of the implanted magnets inside the patient's amputated arm (Fig. 5). The designed FIST & BALL game exercise is developed as an Add-On based on Blender software in the python programming language. At the 3D-View panel of Blender, there is an orange ball, a 3D model of the hand, and nine red circles representing the nine LEDs of the electronic Myo. Quantifying the motion into nine grades, LEDs will turn on one by one, in turn, as the motion progresses. The patient can choose the duration of exercise (2, 4, 6 min) and its difficulty level (easy, medium, and hard), which affect the pace of the moving ball (Fig. 5).

#### Prosthesis characteristics

FUM Bionic hand is designed for people who suffer from hand amputation to enable them to continue their daily tasks. In this prototype, there are five individual motors with worm gears for each finger, letting patients use natural, coordinated grasp patterns with 7 degrees of freedom. The specific design enables independent finger control and absorbs collision forces. Moreover, the actuators are placed in the bionic hand's palm to ease the holding of objects of various shapes.

### Ethical approval and informed consent

This study was approved by the Research Ethics Committee of Mashhad University of Medical Sciences (approval number: IR.MUMS.MEDICAL.REC.1398.099) and is conducted in full compliance with the codes of ethical conduct from the 1964 Declaration of Helsinki. We confirm that informed consent was obtained from our participant (Attachment 1).

## Supplementary Information

Supplementary Video 1.

Supplementary Video 2.

Supplementary Video 3.

Supplementary Video 4.

Supplementary Information 1.

## Data Availability

All data are available in the main text or supplementary materials.
