# Supplementary material for: Clinical implementation of a bionic hand controlled with kineticomyographic signals
Source: Sci Rep. 2022 Aug 31;12:14805. doi: 10.1038/s41598-022-19128-1 (PMC9433417; doi:10.1038/s41598-022-19128-1)
Supplement: Supplementary file 5 — Supplementary Information 1. [file 41598_2022_19128_MOESM5_ESM.pdf]

Supplementary Information

Simulator 1

The magnet localizer system includes three-axis magnetic sensors, a neodymium magnet, and a PC (Fig. S1a). The advantages of selected three-axis sensors include 1) the ability to decrease the number of sensors, and 2) increase the accuracy due to measuring three components of the field. First, the information received from the sensors is sent to the ARM processor by the serial connection. Then, this information is sent to the MATLAB software by the network connection (TCP/IP). The sensor board is fixed horizontally on the base of the system.

Active Noise Cancelation in Simulator 1

One of the ways to decrease or eliminate noise is by adding a sensor to measure noise value and then subtracting it from other magnetic field measurements. To do so, first, the proper location for the noise sensor should be determined. The noise sensor should be as close as possible to the main sensors because the value of noise reported from the noise sensor should be equal to other main sensors. Also, the magnetic field of the implanted magnet should not be measured by the noise sensor since, in this case, the main information will be lost.

Simulator 1 Simulation Results

As shown in Fig. S1b, test setup 1 is used to compare the neural network with the Dipole model in tracking magnets in space. First, the position of the magnet in a zigzag course is used to train the neural network. Afterward, the curved line is followed by the magnet, and the position of the magnet is estimated by both the neural network and Dipole model based on the sensed components of the magnetic field. Table S1 shows the error rate of the neural network and dipole in different arrangements of sensors.

According to these results, about 5 to 7 centimeters distance from the main sensors is acceptable for 2.4×3.6 magnets if the source of the magnetic noise is 2.5 centimeters apart from the main sensors. In this distance, the magnetic fields sensed by the noise sensor are 10 % of the values of the main sensors.

Simulator 2

In this test setup, the performance of magnets as passive sources of the magnetic field in the muscles is simulated (Fig. S1c). Each magnet is mounted on the 304 steel wire, and these wires are driven by a pulley mechanism and a stepper motor. Then, magnetic sensors measure the varying fields caused by linear movements of magnets. Each wire and the magnet attached to it stand for a muscle in the body and implanted magnet.

The Signal Type

The mean, standard deviation, and the correlation of the signal are not constant. In these signals, the movement rate of the tags creates frequency variation, and the addition of the external magnetic field causes a trend in the data. At the same time, the noises created by different apparatus have different magnitudes and standard deviations. In the following table (Table S2), several metrics are applied to measure the properties of the 12 channel signal received from the 12 sensors of the designed socket.

Studying the type of signals indicates that the magnetic noises could easily change the nature of these signals. Therefore, we propose using a filter for moderating the signals. The results of the Hurst exponent show that the current high positive or negative changes are later followed by high negative or positive changes. Furthermore, the low value of Spectral entropy indicates that the irregularity is low and that the signal has repetitive parts. The results of studying the signal match the nature of the motion of the hand and the related muscle contraction and relaxation. In this study, the filter is only applied to the experimental model. As such, future studies could benefit from investigating the design of more developed filters and noise sensors.

Fig. S2 shows the schematic of operation steps. In order to measure the tag displacements, the fluoroscopic films are used to determine the moments when the tags had maximum displacement, which is then compared with the neutral position (Fig. S3).

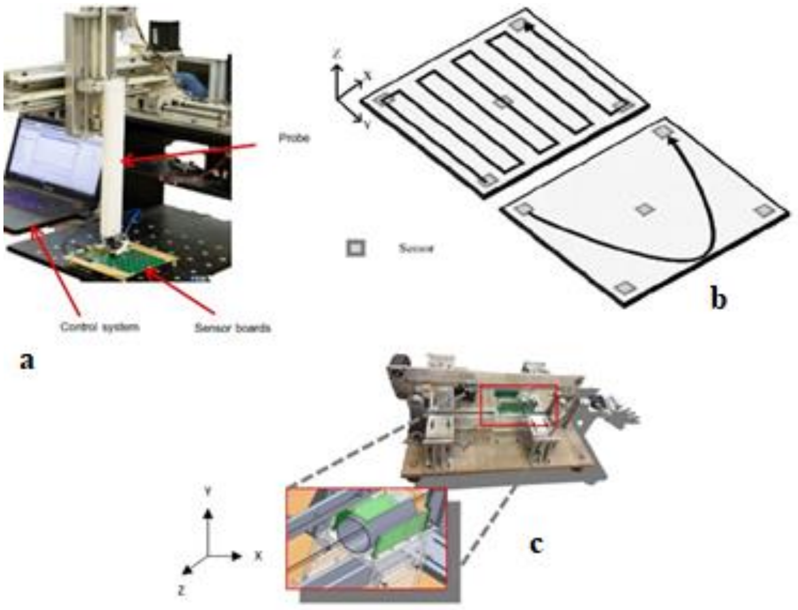

Fig. S1. Simulators of magnets implanted on forearm muscles. (a) Simulator 1: The magnet is attached to the end effector of the three-degree-of-freedom Cartesian robot by a probe fixed to an arm. The received position of the magnet is sent to the MATLAB software through the ARDUINO board by serial communication. In this experiment, magnets travel on the sensor board, which includes 30 sensors. (b) Traveled path by the magnet in the simulator 1. (c) Simulator 2: Each pair of magnet and cable have three degrees of freedom. Movement in the z-axis direction is motor driven while in the x-axis and y-axis are manual.

Table S1. Comparing the neural network with the Dipole model

	Neural network			Dipole model		
	Max error in X axes (mm)	Max error in y axes (mm)	Euclidean distance error (mm)	Max error in x axes (mm)	Max error in y axes (mm)	Euclidean distance error (mm)
5 sensors	5.59	26.018	1.37	3202.7	3261.5	242.819
10 sensors	1.437	0.763	0.114	72.15	25.835	17.75
15 sensors	1.164	0.976	0.078	30.551	5.764	14.847

Table S2. Properties of KMG signal

Metric	Maximum value	minimum value	Mean value
Hurst exponent	0.9959	0.9611	0.9836
Spectral entropy	0.2835	0.2801	0.2813

Designing and Constructing the Socket for MCNN-TG algorithm

One of the challenges of this study is to design a wearable gadget specifically for the arm in a way that the magnetic sensors could measure the magnetic field around the hand. As such, we prepared a 3D print of the patient's amputated stump in order to make his customized socket (Fig. S4).

Referring to the prepared fluoroscopic films, the correct sites for gadget sensors to be placed on the patient's limb socket were determined so the muscle movements are synchronized with the corresponding sensors. The socket apparatus has 4 strip boards, 16 LIS3MDL sensors, one STM32 F030F4 microcontroller, one NRF 24L01, and the main board composed of an STM32 F407VG microcontroller, an NRF24L01, and an ESP8266 WIFI Module. The strip boards collect the data sent by magnetic sensors and forward them to the mainboard. The mainboard sends the

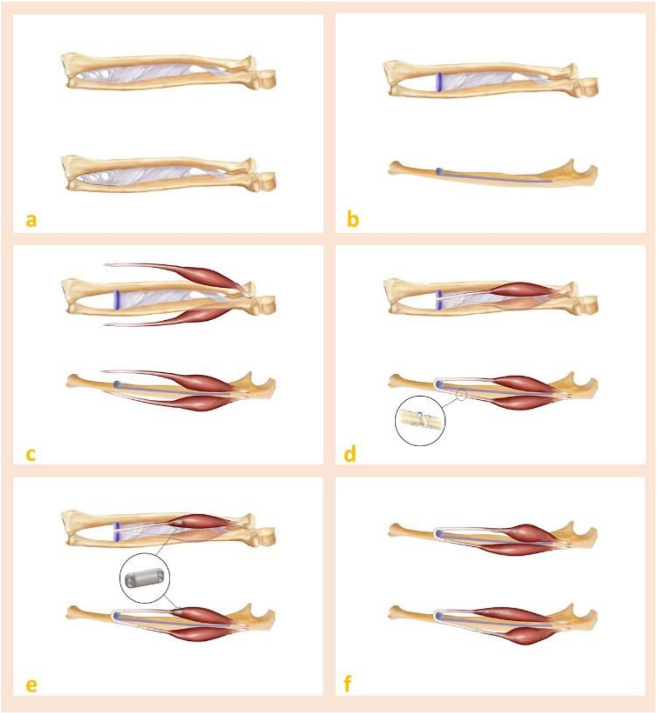

Fig. S2. Schematic illustration of tendon transfers operation technique and tag insertion. Volar and dorsal aspects are exposed to reach the interosseous membrane (a); distal five centimeters of the interosseous membrane are released from its bony attachments (b) and is rolled up proximally to function as a fulcrum for each pair of muscles and the target muscles are released from other adjacent muscles and connective tissue (c); the flexor tendons are transferred to the corresponding extensor tendons under moderate tension (d). We try to place the suture lines far from the fulcrum (d). Magnetic tags are placed in the most superficial flexor level inside the musculotendinous junction (e and f).

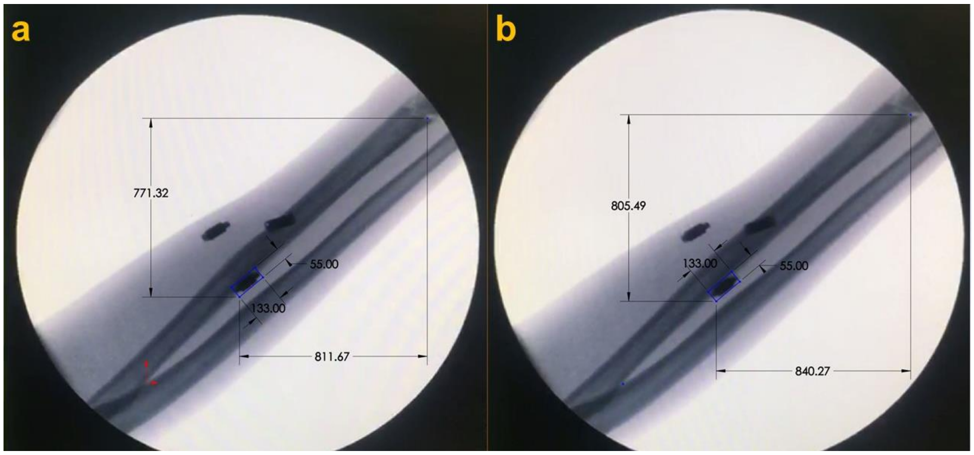

Fig. S3. Measuring the displacement of tag number one in finger flexion and extension movement on fluoroscopic film. Considering the tag length (13.3 mm) as the scale, we measured the maximum displacement of each tag toward the two axes (X & Y) separately during each movement (finger flexion and extension, wrist flexion and extension, and thumb flexion and extension) by using SOLIDWORK software. The neutral tag position (a) and the moment of maximum displacement (b) are compared with each other, and the resultant displacement magnitude ($\sqrt{x^2 + y^2}$) is reported in Table 1.

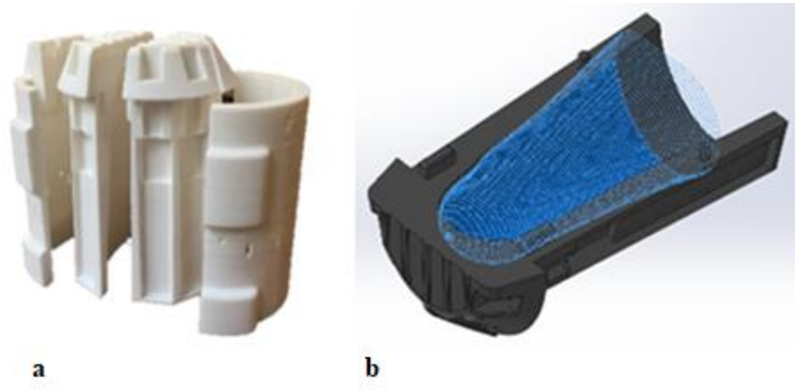

Fig. S4. Socket (a) and 3D scan (b) of the arm.

controlled data to the MCNN-TG algorithm in MATLAB software to be processed and displayed. The inner design of our gadget is based on three-dimensional scanning to facilitate the repeatability of the sensor's location in different instances of data registering and to secure and stabilize the gadget's position. Our goals for designing this gadget are: 1) the optimal localizing of the sensors and determining the studying minimum number of sensors needed for reaching the desired performance, and 2) studying the displacement of magnets compared to each other in different movements.

Rehabilitation Game Exercise

The importance of rehabilitation exercises is undeniable to make the patients more confident to perform specific gestures, e.g. making a fist with the help of bionic hands, in their daily life. Also, these exercises should be motivating enough to be followed by the patients and make them aware of their progress. For this purpose, we have implemented a rehabilitation game exercise in which the given objective for the patient is to catch and keep the ball which is moving up and down with the 3D model of a hand in a virtual environment through movements of the implanted magnets inside the patient’s amputated arm (Fig. 5).

When the patient runs the game, the orange ball moves down perpendicular to the palm of the 3D model of the hand in the Z-Axis direction. As soon as the ball reaches the surface of the hand, its direction will be reversed and it moves up to its starting point. While the ball continues its periodic up and down movements, the patient should catch and hold it when it is near to the palm until the end of the exercise. The overall time the patient keeps the ball is measured and reported to the patient. Also, all the preferred options of the game and the progress of the patient during the game will be saved to a separate file which can later be reviewed by the patient.
